# Emotional intelligence as a predictor of functional outcomes in psychotic disorders

**DOI:** 10.1016/j.schres.2025.01.005

**Published:** 2025-01-25

**Authors:** Jennifer M. Blank, Roman Kotov, Katherine G. Jonas, Wenxuan Lian, Elizabeth A. Martin

**Affiliations:** a University of California, Irvine, Department of Psychological Science, 4102 Social and Behavioral Sciences Gateway, Irvine, CA 92617, United States; b Stony Brook University, Department of Psychiatry & Behavioral Health, HSC T10 060, Stony Brook, New York 11794, United States

**Keywords:** MSCEIT, Social cognition, Social performance, Functional impairment

## Abstract

Psychotic disorders are associated with significant impairment in psychosocial functioning, yet mechanisms associated with this impairment remain poorly understood. Emotional intelligence, a component of social cognition, is associated with psychosocial functioning in this population. However, prior work has used relatively small samples, reported inconsistent relations between functioning domains and emotional intelligence, and inconsistently considered negative symptoms. To address these limitations, we examined the predictive ability of emotional intelligence on functional outcomes using a five-year longitudinal design. We used a large sample of individuals with and without psychotic disorder diagnoses (N = 324), a performance-based measure of emotional intelligence, and three measures of functioning (i.e., social performance, assessor-rated social and occupational functioning, self-rated functioning in independent living). Results revealed individuals diagnosed with a psychotic disorder have lower emotional intelligence than those without a history of psychosis. Emotional intelligence was associated with social performance and social and occupational functioning in both those with and without a history of psychosis. In those diagnosed with a psychotic disorder, emotional intelligence and negative symptoms better predict social performance (*β*_Emotional_ = 0.36, ${\text{R}}_{\text{delta}}^{2}=0.09$) and social and occupational functioning (*β*_Emotional_ = 0.21, R^2^ = 0.03), but not self-rated functioning in independent living (*β*_Emotional_ = −0.08, ${\text{R}}_{\text{delta}}^{2}=0.00$), as compared to negative symptoms alone. Overall, findings support the use of emotional intelligence as a longitudinal predictor of social and occupational outcomes above and beyond negative symptoms alone. This work highlights potential, specific intervention targets for individuals with psychotic disorders.

## 1. Introduction

Psychotic disorders are a leading cause of disability, largely due to deficits in psychosocial functioning (Harvey et al., 2012; Świtaj et al., 2012; Vos et al., 2017). Research has attempted to better understand determinants of functional impairments in this population to identify mechanisms that can be targeted to improve related outcomes. Due to the critical role that social processes (e.g., social connection) play on physical and mental health outcomes (Holt-Lunstad, 2023), paired with the significant impairment in social functioning found in this population, one area of focus that has gained attention is social cognition (Green et al., 2008). Investigating the relation between social cognition, symptoms associated with psychotic disorders, and functioning may allow us to better identify targetable change mechanisms. An aim of this study was to examine whether emotional intelligence, a key domain of social cognition, predicts functional outcomes five years later in a sample of individuals with psychotic disorder diagnoses.

Social cognition refers to the processes and mechanisms of obtaining, organizing, and responding to information in one’s environment related to social settings and interactions (Frith, 2008). Research on social cognition has consistently found impairment in individuals with schizophrenia compared to non-affected individuals (see Penn et al., 2008 for a review). Abnormalities in social cognition are further associated with symptoms—particularly negative symptoms (Ventura et al., 2013; Yolland et al., 2021)—along with a range of functional outcomes (e.g., social skills, community functioning, independent living; Couture et al., 2006; Fett et al., 2011; Halverson et al., 2019). Yet, social cognition is a broad construct consisting of several domains (e.g., emotional intelligence, theory of mind, attributional style, social knowledge and perception; Green et al., 2008), resulting in some inconsistencies in these findings. One domain that has been frequently studied in individuals with psychotic disorders, consistently associated with functional outcomes, and shown to be amendable to treatment (Tan et al., 2018) is emotional intelligence.

Emotional intelligence is the ability to recognize, understand, discriminate among, and respond to one’s own and others’ emotions (Salovey and Mayer, 1990). Cross-sectional work reveals higher levels of emotional intelligence to be associated with positive outcomes in children and adults across settings (e.g., school, work, home) and domains (e.g., relationship quality, psychological and physical well-being; see Mayer et al., 2008 for a review). Individuals with psychosis experience significant impairments in emotional intelligence (Martins et al., 2019; Savla et al., 2013), which has been found to correlate with reductions in social, community, and independent functioning in cross-sectional studies (Kee et al., 2009; Kimhy et al., 2012; Lin et al., 2013). Overall, this work demonstrates that emotional intelligence is one aspect of social cognition that has been consistently linked to functional impairment in both those with and without a history of psychosis. However, it remains unclear whether the strength of this association is comparable across these two groups, particularly when using the same measures of functioning. As such, a goal of this study is to test whether emotional intelligence similarly predicts functioning in those with and without a history of psychosis to better understand the nature of this relation.

In addition to cross-sectional evidence of the link between emotional intelligence and functional outcomes of psychosis, early longitudinal studies have provided preliminary evidence that emotional intelligence predicts functioning one to two years later (Eack et al., 2011 [N = 58]; Hoe et al., 2012 [N = 105]; Horan et al., 2012 [N = 55]; Kee et al., 2003 [N = 81]). To date, only one study has used a longer follow-up period, reporting emotional intelligence to be associated with community functioning five years later in those with psychotic disorders (N = 41; McCleery et al., 2016). Of this work, most used a composite score to measure global functioning (Eack et al., 2011; Hoe et al., 2012; McCleery et al., 2016). Of those studies that assessed multiple domains of functioning, emotional intelligence was found to differentially predict these domains (e.g., emotional intelligence as a stronger predictor of occupational functioning than social functioning; Horan et al., 2012, Kee et al., 2003). Further, some of this work has found inconsistent relations between functional domains and emotional intelligence (e.g., correlations between baseline emotional intelligence and follow-up social functioning ranging from *r* = 0.04 to 0.34; Hoe et al., 2012; Horan et al., 2012). As such, it is unclear how emotional intelligence predicts different domains of functioning several years later in individuals with psychotic disorders.

Negative symptoms are also associated with functioning in individuals with psychosis (Harvey and Strassnig, 2012; Hunter and Barry, 2012) but inconsistently associated with emotional intelligence (e.g., correlations between emotional intelligence and negative symptoms ranging from 0.002 to 0.43; Bell et al., 2013; DeTore et al., 2018; Eack et al., 2010; Kee et al., 2009; Lin et al., 2013; McCleery et al., 2016). This work suggests that impairments in emotional intelligence are related to, yet not redundant with, negative symptoms. These studies did not, however, provide information on whether emotional intelligence has added utility in predicting functional outcomes in this population above and beyond negative symptoms. Therefore, this study aims to further examine the association between emotional intelligence and negative symptoms in a larger sample of individuals with psychotic disorders and test whether negative symptoms impact the relation between emotional intelligence and different functional domains.

Overall, this study aims to address limitations of extant literature examining longitudinal relations between emotional intelligence and negative symptoms with functional outcomes in individuals with psychotic disorders. To accomplish this, we used a five-year longitudinal design, a large sample of individuals with and without psychotic disorder diagnoses (N = 324), a performance-based measure of emotional intelligence, and three measures of functioning (i.e., social performance, assessor-rated social and occupational functioning, self-rated functioning in independent living). Based on extant literature, we hypothesized those with psychotic disorders would have lower emotional intelligence than those without a history of psychosis. We also hypothesized that emotional intelligence would predict each of the three functional outcomes five years later in both those with and without psychotic disorders. Further, we hypothesized that negative symptoms would be associated with emotional intelligence and each of the functional outcomes measured five years later in those with psychotic disorders. Exploratory analyses were employed to examine whether the strength of the association between emotional intelligence and each of the functional outcomes differed in those with a psychotic disorder, as well as to examine whether psychotic disorder status moderated the association between emotional intelligence and each of the functional outcomes.

A second aim of this study was to test whether emotional intelligence predicts each of the functional outcomes measured five years later over and above negative symptoms in those with psychosis. We hypothesized that emotional intelligence and negative symptoms would better predict social performance, assessor-rated social and occupational functioning, and self-reported functioning in independent living five years later than negative symptoms alone in those with psychotic disorders.

## 2. Methods

### 2.1. Participants

This study used data from the Suffolk County Mental Health Project, an ongoing, longitudinal, cohort study following individuals with a first-episode of psychosis for 25+ years (Bromet et al., 1992; Bromet et al., 2011). Participants were recruited between 1989 and 1995 from all 12 inpatient facilities across Suffolk County, New York. Inclusion criteria for this study included a first admission for psychosis within 6 months of the time of enrollment, age 15–60 years, ability to speak English, an IQ above 70, and onset of psychosis unrelated to an apparent medical issue. Written consent (or assent and parental written consent for minors) was collected from all participants. The study was approved annually by the institutional review boards of Stony Brook University and the participating hospitals. Emotional intelligence was added to the study at Year 20 and the most recently completed follow-up time point to date is Year 25. As such, data from Years 20 and 25 were used in the current study.

A total of 628 individuals with a history of psychosis met initial inclusion criteria for the project. Of this sample, 402 did not complete the measure of emotional intelligence at Year 20 for the following reasons: 71 due to time constraints, 86 participated in a modality (e.g., phone interview) which made completion of the measure infeasible, 77 refused to participate, 9 moved abroad and could not be assessed, 6 were too ill to participate, 88 had died, and 65 were lost to follow-up. Of the 226 participants with available emotional intelligence data, by Year 25 an additional 30 participants had died and 53 were lost to follow-up, resulting in a final sample of 143 individuals with a history of psychosis (i.e., “psychosis group”). Attrition analyses revealed participants not included in the current study were slightly older and lower on inexpressivity assessed at baseline than those included in the study (see Supplementary materials, Table 1 for full attrition analyses).

This study also included participants without a history of psychosis. These participants—matched on age and sex—were recruited at Year 20 using random digit dialing within the zip codes in which the original first-admission sample was recruited. Exclusion criteria included a history of psychosis or psychiatric hospitalization (to further mitigate inclusion of participants with a history of psychotic features). A total of 261 individuals qualified for the study. Of those, 80 participants didn’t complete the emotional intelligence measure or at least one of the functional outcome measures due to time constraints, yielding a final sample of 181 individuals without a history of psychosis (i.e., “never-psychotic group”). Attrition analyses revealed no differences in demographic or clinical variables between those included and those not included in the current study (see Supplementary materials, Table 1).

### 2.2. Measures

#### 2.2.1. Emotional intelligence

Emotional intelligence was measured at Year 20 using the Mayer-Salovey-Caruso Emotional Intelligence Test (MSCEIT; Mayer, 2002), a widely used performance-based tool of emotional intelligence. The MSCEIT consists of four branches: Perceiving Emotions, Facilitating Thought, Understanding Emotions, and Managing Emotions. Of the MSCEIT battery, only the Managing Emotions branch was administered to participants; the other three branches were not. The Managing Emotions branch has been found to correlate more strongly with functional outcomes than the other three branches (Nuechterlein et al., 2008). As such, this branch has been recommended by the National Institute of Mental Health and included in the MATRICS Consensus Cognitive Battery (Green et al., 2005), a standardized neurocognitive battery used in research with individuals with schizophrenia. The Managing Emotions branch consists of two tasks, emotion management and social management. The emotion management task assesses one’s ability to regulate their own emotions in difficult situations. Participants are asked to read hypothetical scenarios and rate the effectiveness of different actions in achieving a certain outcome. The social management task is similar, but assesses an individual’s ability to incorporate their own and others’ emotions into decision making. The Managing Emotions branch of the MSCEIT has been found to have strong internal consistency in individuals with a schizophrenia diagnosis (*α* = 0.81; Eack et al., 2010), good test-retest reliability, and utility as a repeated measure (Green et al., 2005).

#### 2.2.2. Functioning

Three facets of functioning were measured five years after the assessment of emotional intelligence—social performance, assessor-rated social and occupational functioning, and self-rated functioning in independent living. Social performance was measured using the Social Skills Performance Assessment (SSPA; Patterson et al., 2001). The SSPA is a task-based assessment adapted from Bellack et al., 1990 to assess social skills in individuals with schizophrenia. Participants are asked to role-play two different scenarios with the interviewer (i.e., introducing themselves to a new neighbor, requesting their landlord fix a leak). Participants’ recorded role plays are rated on several domains (e.g., speech fluency, clarity, focus, negotiating ability) using a 5-point Likert scale (higher scores indicate greater social skills). Role plays were scored by an expert rater considered to be the gold standard for SSPA ratings. The SSPA is widely used with populations with psychosis and has been found to have good psychometric properties (Miller et al., 2021).

Assessor-rated social and occupational functioning was assessed using the Social and Occupational Functioning Assessment Scale (SOFAS; Goldman et al., 1992). The SOFAS assesses functioning on a scale of 0 to 100, with anchors at each 10-point increment to facilitate scoring. Scores were determined through consensus among study psychiatrists. The SOFAS considers functioning across several domains (e.g., work, relationships, daily activities, leisure activities) and has been found to be both valid and reliable, including in populations with psychosis (Goldman et al., 1992; Hilsenroth et al., 2000).

The 12-item version of the World Health Organization Disability Assessment Schedule (WHODAS2.0; Üstün et al., 2010) was used to assess self-reported functioning. The WHODAS asks participants to rate the level of difficulty they’ve had completing several tasks of daily independent living (e.g., taking care of household responsibilities, getting dressed) over the past 30 days on a scale of 1 (None) to 5 (Extreme or cannot do). Participants are also asked to report the number of days they were unable to carry out their normal activities due to these difficulties. Higher scores reflect greater levels of difficulty. The WHODAS is a widely used measure of disability, replacing the Global Assessment of Functioning in the DSM-5.

#### 2.2.3. Symptom dimensions

Negative symptom severity was assessed using the Scale for the Assessment of Negative Symptoms (SANS; Andreasen, 1983) and the Scale for the Assessment of Positive Symptoms (SAPS; Andreasen, 1984). The SANS consists of 20 items assessing negative symptoms across 5 domains (i.e., affective flattening or blunting, alogia, avolition/apathy, anhedonia/asociality, and attention), while the SAPS consists of 30-items assessing positive symptoms across 4 domains (i.e., hallucinations, delusions, bizarre behavior, and positive formal thought disorder). Kotov et al. (2016) conducted a factor analysis of the SANS and SAPS and found a four-factor structure (i.e., positive/reality distortion, disorganization, inexpressivity, and avolition) to best explain variation in both neural markers and long-term outcomes related to functioning. Thus, in the current study, inexpressivity and avolition were used as measures of negative symptoms, with higher scores indicating greater symptom severity.

### 2.3. Statistical plan

Pearson’s chi-square tests and independent samples *t*-tests were used to test for demographic differences between the psychosis and never-psychotic groups. An independent samples *t*-test and analysis of covariance (ANCOVA) were employed to test for differences in emotional intelligence across groups, with the latter controlling for relevant demographic variables (i.e., education, employment status). Zero-order correlations were used to test the associations between emotional intelligence, negative symptoms, and functional outcomes. Steiger’s *Z*-tests (Steiger, 1980) were used to compare correlation strengths between emotional intelligence, social performance, social and occupational functioning, and functioning in independent living. Because of missing data, sample sizes varied across correlations, which may impact the reliability of this test. As such, *Z*-tests were only performed on those with complete data (N = 59). Moderation analyses were employed to examine whether the relation between emotional intelligence and each of the functional outcomes varied as a function of group status. For moderation analyses, emotional intelligence was mean centered and group status was dummy coded with the never-psychotic group coded as the reference group. To test the robustness of findings, models were also run controlling for relevant demographic variables (e.g., age, education, employment status).

Three hierarchical regression models were employed to test whether emotional intelligence and negative symptoms at Year 20 better predict functional outcomes five years later for the psychosis group than negative symptoms alone. For each regression model, negative symptoms (i.e., avolition and inexpressivity) were entered into the model first (step 1), followed by emotional intelligence (step 2), and each functional outcome was included as the criterion. Models were also run controlling for relevant demographic variables (i.e., age, education, employment status) and negative symptoms assessed at Year 25 to test the robustness of findings. Each of these regressions were powered at 0.80 to detect a medium effect size of *R*^*2*^ = 0.14 (Faul et al., 2007). All analyses were conducted using IBM SPSS Statistics Version 29 (IBM Corp. Released, 2023). We applied a false discovery rate (FDR; Thissen et al., 2002) correction to all planned analyses (i.e., correlations, Steiger’s *Z*-tests, moderations, hierarchical regressions).

## 3. Results

### 3.1. Group differences

Sample characteristics for the psychosis and never-psychotic groups, along with group differences, can be found in Table 1. Groups differed on age and educational attainment, as well as employment status at both Year 20 and Year 25. The psychosis group scored lower on social performance (as measured by the SSPA) and assessor-rated social and occupational functioning (as measured by the SOFAS) and higher on self-rated functioning in independent living (as measured by the WHODAS) as compared to the never-psychotic group.

As hypothesized, the two groups differed on level of emotional intelligence, with the psychosis group scoring significantly lower than the never-psychotic group (Cohen’s *d* = −0.67; see Table 1). Because emotional intelligence was found to vary as a factor of education level [*F*(7,315) = 8.67, *p* < .001, Cohen’s *f* = 0.41] and employment status [*F*(3,320) = 15.68, *p* < .001, Cohen’s *f* = 0.37], group differences in emotional intelligence were also tested controlling for these two variables. The pattern of findings persisted, with those in the psychosis group scoring significantly lower on emotional intelligence than the never-psychotic group [*F*(1,319) = 10.467, *p* = .001; Cohen’s *f* = 0.17].

### 3.2. Association between emotional intelligence, functioning, and negative symptoms across groups

Table 2 displays the correlations between emotional intelligence, negative symptoms, and functional outcomes for both the psychosis and never-psychotic groups. In partial support of our hypotheses, emotional intelligence had small-to-moderate, statistically significant associations with social skills performance (SSPA) and assessor-rated social and occupational functioning (SOFAS) at follow-up in the never-psychotic group, but a small and non-significant association with self-rated functioning of independent living (WHODAS). For the psychosis group, both emotional intelligence and negative symptoms had significant small-to-large associations with each functional outcome variable assessed five years later, with the exception of the WHODAS, which was not significantly associated with emotional intelligence. Across both groups, functional outcomes had small-to-moderate correlations with one another, demonstrating that these outcomes are related, but not redundant. In the psychosis group, both social performance and self-reported functioning in independent living were moderately correlated with assessor-rated social and occupational functioning, but weakly correlated with each other. Steiger’s *Z*-tests revealed that, in the psychosis group, emotional intelligence was more strongly related to assessor-rated social and occupational functioning than self-reported functioning of independent living (*z* = 3.86, *p* ≤ 0.001). Emotional intelligence was also more strongly associated with social performance than self-reported functioning of independent living (*z* = 2.32, *p* = .020), but this finding did not withstand the FDR correction. The strength of the association between emotional intelligence and social and occupational functioning was not found to be statistically significantly different from the association between emotional intelligence and social performance (*z* = −1.06, *p* = .289). Adjusted correlation coefficients used for Steiger’s *Z*-tests can be found in the Supplementary materials (Table 2).

**Fig. 1.**
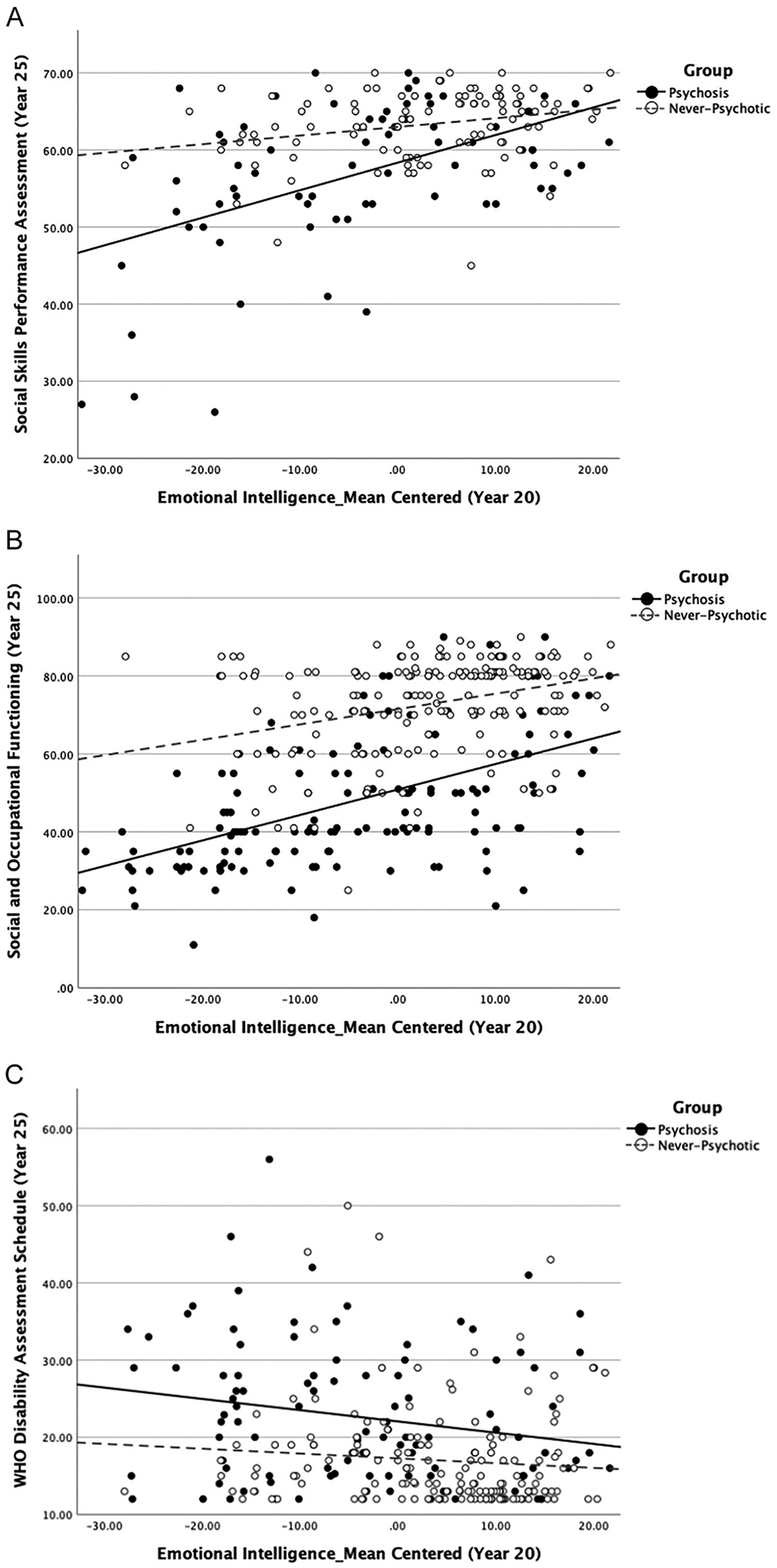
Group status as a moderator of the relation between emotional intelligence and functioning. A. Emotional intelligence and social performance. Interaction: *b* = 0.24, 95 % CI (0.08, 0.41), *t*(169) = 2.94, *p* = .004, β = 0.28. Psychosis Group: *b* = 0.36, 95 % CI (0.25, 0.47), *t*(169) = 6.50, *p* < .001, β = 0.56. Never-Psychotic Group: *b* = 0.11, 95 % CI (−0.01, 0.24), *t*(169) = 1.80, *p* = .074, β = 0.18. B. Emotional intelligence and assessor-rated social and occupational functioning. Interaction: *b* = 0.26, 95 % CI (0.00, 0.52) t(320) = 2.00, *p* = .047, β = 0.12. Psychosis Group: *b* = 0.65, 95 % CI (0.49, 0.82), *t*(320) = 7.82, *p* < .001, β = 0.41. Never-Psychotic Group: *b* = 0.39, 95 % CI (0.20, 0.59), *t*(320) = 3.93, *p* < .001, β = 0.25. **Note.** Interaction is no longer statistically significant after applying the FDR correction. C. Emotional intelligence and self-rated functioning in independent living. Interaction: *b* = −0.08, 95 % CI (−0.25, 0.08), *t*(263) = −1.00, *p* = .319, β = −0.09. Psychosis Group: *b* = −0.15, 95 % CI (−0.26, −0.03), *t*(263) = −2.57, *p* = .011, β = −0.21. Never-Psychotic Group: *b* = −0.06, 95 % CI (−0.19, 0.06), *t*(263) = −0.99, *p* = .325, β = −0.09. **Note.** Higher scores indicate greater functional impairment.

Group status significantly moderated the association between emotional intelligence and social performance at follow-up, such that the positive association between emotional intelligence and social performance was stronger for the psychosis group than for the never-psychotic group (see Fig. 1a). Group status was not a significant moderator of the association between emotional intelligence and assessor-rated social and occupational functioning or self-rated functioning of independent living (see Fig. 1b and c). To test the robustness of these findings, analyses were ran controlling for age, education attainment, and employment status. Findings persisted with similar effect sizes after controlling for these variables (see Supplementary materials, pp. 3).

### 3.3. Emotional intelligence as a predictor of functional outcomes controlling for negative symptoms

Table 3 displays the results from the hierarchical regression models for the psychosis group. In partial support of our hypotheses, emotional intelligence and negative symptoms better predict both social performance and assessor-rated social and occupational functioning than negative symptoms alone. However, emotional intelligence and negative symptoms do not better predict self-rated functioning in independent living five years later than negative symptoms alone. Findings were robust, with the same patterns and similar effect sizes persisting after controlling for relevant demographic variables (see Supplementary materials, Tables 3, 4, and 5) and after adding negative symptoms assessed at Year 25 to the model (see Supplementary materials, Table 6).

## 4. Discussion

The current study used a longitudinal design and large sample of individuals with and without psychotic disorders to test the relation between emotional intelligence and different domains of functioning five years later (i.e., social performance domain [SSPA], social and occupational functioning domain [SOFAS], independent living domain [WHODAS]). This is the first study to directly compare association strengths between emotional intelligence and functional outcomes years later across a group of individuals with psychotic disorder diagnoses and a group of individuals without a history of psychosis. In line with extant work, the psychosis group scored lower on emotional intelligence than the never-psychotic group (Martins et al., 2019; Savla et al., 2013). Further, emotional intelligence was significantly and positively associated with both social performance and assessor-rated social and occupational functioning five years later across both groups. This provides evidence that emotional intelligence predicts some aspects of functioning in populations both with and without a history of psychosis years later.

Emotional intelligence was significantly more predictive of social performance in the psychosis group than in the never-psychotic group. Similarly, emotional intelligence was more predictive of social and occupational functioning in the psychosis group than in the never-psychotic group, although this finding did not reach statistical significance. This may suggest that mechanisms underlying the relation between emotional intelligence and functioning differ between those with and without psychotic disorders. Specifically, our findings may reflect a unique relation between emotional intelligence and social performance in those with a history of psychosis, such that impairment found in both in those with psychotic disorders are due to a shared underlying mechanism, suggesting that emotional intelligence may be an important treatment target in this population. Future work could examine whether emotional intelligence differentially predicts functioning in those with psychotic disorders as compared to other clinical populations without a history of psychosis (e.g., Major Depressive Disorder) to determine whether the associations observed in the current study are transdiagnostic or unique to the experience of psychosis.

A large body of extant work has examined associations between different facets of social cognition and functioning in individuals with psychosis. Most of this work has been cross-sectional and has found association strengths ranging from *r* = 0.20 to 0.30 (Fett et al., 2011; Halverson et al., 2019). Longitudinal work in those with early psychosis have found larger associations between social cognition domains and functioning (*r*_*Pooled*_ = 0.43, 95 % CI [0.29–0.56]; Cowman et al., 2021). Our study found similar sized associations between emotional intelligence–one domain of social cognition–and different domains of functioning five years later (|*r*| = 0.21–0.51). These findings provide support that emotional intelligence–as measured by the Managing Emotions branch of the MSCEIT–is one domain of social cognition that may equally or better predict some functional outcomes in this population as compared to other social cognition domains. Future work could further test this hypothesis.

As hypothesized and consistent with prior work, negative symptoms were found to be associated with impaired functioning years later in the psychosis group (Harvey and Strassnig, 2012; Hunter and Barry, 2012). While extant work has found negative symptoms to be inconsistently associated with emotional intelligence (Bell et al., 2013; DeTore et al., 2018; Eack et al., 2010; Kee et al., 2009; Lin et al., 2013; McCleery et al., 2016), both inexpressivity and avolition had moderate associations with emotional intelligence (*r* = −0.47 and −0.34, respectively) in the current study. As such, negative symptoms were included in the hierarchical regression model so as to allow us to test whether emotional intelligence has utility in predicting functioning five years later above and beyond negative symptoms. Emotional intelligence and negative symptoms more strongly predicted social performance and assessor-rated social and occupational functioning than negative symptoms alone. These findings provide evidence that emotional intelligence has added utility in predicting functional outcomes in this population above and beyond negative symptoms.

The current study used both an assessor-rated and a self-report measure of functional impairment, finding emotional intelligence to be more strongly associated with observer-rated functioning in the psychosis group. While previous work suggests that individuals with psychotic disorders both overestimate and underestimate their functional abilities compared to other sources (e.g., family members, clinicians; Harvey and Pinkham, 2015), we found a moderate association (*r* = −0.43) between these two assessments (although not large enough to be redundant). This moderate-sized relation is similar to that found by Sabbag et al. (2011) on some functional outcomes (i.e., Social Functioning Scale, Quality of Life Scale). However, the current study used two distinct measures of assessor- and self-rated functioning. It is therefore possible that the WHODAS more closely aligns with assessor-rated functioning than other scales.

### 4.1. Limitations

The sample used in this study consisted of individuals with a chronic psychotic disorder. As such, we are unable to conclude whether emotional intelligence similarly predicts functioning in individuals with early psychosis or at risk for developing psychosis. While some work suggests emotional intelligence predicts community functioning in those in the early stages of illness and those with chronic psychosis (McCleery et al., 2016), future work should test whether this pattern persists across other domains of functioning. Additionally, extent work reveals that earlier onset of psychotic disorders is associated with worse outcomes (e.g., negative symptoms, social and occupational functioning, global outcomes; Immonen et al., 2017). Therefore, future work should test whether the findings from this study replicate across different developmental stages of psychosis.

Additionally, a strong association was found between emotional intelligence and social performance five years later (*r* = 0.51). Because these constructs were not measured at the same wave, we were unable to test their cross-sectional relations. As such, it remains unclear whether these assessments are capturing a similar underlying process, or whether emotion regulation in the self and others (MSCEIT) influences social skill proficiency (SSPA). While limited work suggests these constructs are distinct (Corbera et al., 2013), future work should further examine this relation.

## 5. Conclusion

The current study advances our understanding of the relation between emotional intelligence and functional outcomes both in individuals with and without a history of psychosis. This study is the first to demonstrate that emotional intelligence more strongly predicts some functional outcomes (i.e., social performance) in those with a psychotic disorder than those without a history of psychosis years later. This study also revealed emotional intelligence to be more strongly associated with assessor-rated social and occupational functioning than self-rated functioning in independent living in those diagnosed with a psychotic disorder. Finally, we found emotional intelligence to predict some functional domains (i.e., social performance [SSPA], social and occupational functioning [SOFAS]) over and above negative symptoms alone in those with psychosis. Taken together, these findings support emotional intelligence as one domain of social cognition that can be used to predict social functioning in individuals with psychotic disorders years later. Relatedly, this work suggests that emotional intelligence may serve as a target for intervention for those experiencing impairment related to social dysfunction.

## Supplementary Material

1

## Figures and Tables

**Table 1:** Sample characteristics.

	Psychosis (N = 143)	Never-psychotic (N = 181)	Group differences	Effect size
	M (SD)	M (SD)		
Age (Year 20)	46.97 (8.32)	50.86 (9.08)	*t*(321) = −3.97**	*d* = −0.44
Gender	**N (%)**	**N (%)**	*x*^*2*^(1, N = 323) = 0.89	*V* = 0.05
Male	83 (58 %)	95 (53 %)		
Female	60 (42 %)	85 (47 %)		
Race			*x*^*2*^(4, N = 323) = 3.29	*V* = 0.10
White	117 (82 %)	158 (87 %)		
Asian or Asian American	3 (2 %)	1 (1 %)		
African American or Black	13 (9 %)	11 (6 %)		
Multiracial	5 (4 %)	6 (3 %)		
Other/unknown	5 (4 %)	4 (2 %)		
Education			*x*^*2*^(7, N = 323) = 30.93**	
Less than high school	15 (11 %)	4 (2 %)		
High school degree/GED	80 (56 %)	75 (41 %)		
Associate’s or Bachelor’s degree	41 (29 %)	65 (36 %)		
Master’s degree or equivalent	6 (4 %)	32 (18 %)		
Doctoral degree	1 (1 %)	4 (2 %)		
Employment				
Year 20			*x*^*2*^(3, N = 324) = 63.37**	*V* = 0.44
Full-time work/student	33 (23 %)	114 (63 %)		
Part-time work/student	22 (15 %)	23 (13 %)		
No work/not a student	86 (60 %)	37 (20 %)		
Other or missing	2 (1 %)	7 (4 %)		
Year 25			*x*^*2*^(3, N = 323) = 55.31**	*V* = 0.41
Full-time work/student	32 (22 %)	110 (61 %)		
Part-time work/student	23 (16 %)	23 (13 %)		
No work/not a student	87 (61 %)	45 (25 %)		
Other or missing	1 (1 %)	3 (2 %)		
Diagnosis (Year 20)				
SSD	77 (54 %)	–		
BP with psychotic features	40 (28 %)	–		
MDD with psychotic features	12 (8 %)			
Drug-induced psychosis	7 (5 %)	–		
Other	7 (5 %)			
Medication use				
Year 20				
Antipsychotic	90 (63 %)	2 (1 %)	*x*^*2*^(1, N = 322) = 148.86**	*V* = 0.68
Mood stabilizer	34 (24 %)	0 (0 %)	*x*^*2*^(1, N = 322) = 47.58**	*V* = 0.38
Antidepressant	60 (42 %)	18 (10 %)	*x*^*2*^(1, N = 322) = 44.08**	*V* = 0.37
Year 25				
Antipsychotic	86 (60 %)	2 (1 %)	*x*^*2*^(1, N = 317) = 141.73**	*V* = 0.67
Mood stabilizer	31 (22 %)	1 (1 %)	*x*^*2*^(1, N = 317) = 40.10**	*V* = 0.36
Antidepressant	54 (38 %)	26 (14 %)	*x*^*2*^(1, N = 317) = 23.63**	*V* = 0.27
**Emotional intelligence and functional outcomes**	**M (SD)**	**M (SD)**		
Emotional intelligence (Year 20)	91.32 (13.35)	99.05 (9.91)	*t*(254.51) = −5.78**	*d* = −0.67
SSPA (Year 25)	57.14 (9.75)	63.29 (4.68)	*t*(98.12) = −5.02**	*d* = −0.85
SOFAS (Year 25)	48.08 (17.39)	72.85 (12.28)	*t*(245.79) = −14.43**	*d* = −1.68
WHODAS (Year 25)	22.51 (8.95)	17.04 (6.75)	*t*(189.30) = 5.42**	*d* = 0.71
**Covariates**				
Year 20				
Inexpressivity	7.95 (9.61)	0.86 (2.63)	*t*(155.21) = 8.49**	*d* = 1.07
Avolition	12.34 (8.92)	2.79 (3.89)	*t*(184.59) = 11.94**	*d* = 1.45

Note. Symptoms and markers of functioning were assessed separately from diagnoses.

SSD = Schizophrenia spectrum disorder; BP = Bipolar disorder; MDD = Major depressive disorder; SSPA = Social Skills Performance Assessment; SOFAS = Social and Occupational Functioning Assessment Scale; WHODAS = World Health Organization Disability Assessment Schedule.

*d* = Cohen’s d; *V* = Cramer’s V.

** Indicates a difference between groups at *p* < .001.

**Table 2:** Correlations between emotional intelligence, functional outcomes, and negative symptoms by group.

	Emotional Intelligence (Year 20)	Inexpressivity (Year 20)	Avolition (Year 20)	SSPA (Year 25)	SOFAS (Year 25)	WHODAS (Year 25)
Emotional Intelligence (Year 20)		−.18*	−.13	.25	.32**	−.09
Inexpressivity (Year 20)	−.34**		.14	−.22	−.15	.17
Avolition (Year 20)	−.47**	.58**		−.24*	−.47**	.22*
SSPA (Year 25)	.51**	−.41**	−.46**		.28**	−.16
SOFAS (Year 25)	.50**	−.40**	−.73**	.44**		−.49**
WHODAS (Year 25)	−.21	.03	.36**	−.14	−.43**	

Note. Highlighted cells indicate correlations for the psychosis group. Non-highlighted cells indicate correlations for the never-psychotic group.

Sample sizes differ across correlations due to missing data.

SSPA = Social Skills Performance Assessment; SOFAS = Social and Occupational Functioning Assessment Scale; WHODAS = World Health Organization Disability Assessment Schedule.

* *p* < .05,

** *p* < .001;

Asterisks indicate statistically significant findings that persist after applying the FDR correction.

**Table 3:** Regression coefficients for predictors of functional outcomes in the psychosis group.

Block	Variable	b	95 % CI	SE	*β*	*t*	*p*	R^2^ (Adj. R^2^)	*Δ*F-statistic (df1, df2)
Social performance (SSPA)									
1	Constant	63.48	[60.02, 66.93]	1.73	–	36.65	<0.001	0.24	
	Inexpressivity (Year 20)	−0.24	[−0.50, 0.03]	0.13	−0.22	−1.77	0.081	(0.22)	
	Avolition (Year 20)	−0.38	[−0.66, −0.10]	0.14	−0.33	−2.67	0.009		
2	Constant	36.94	[18.89, 55.00]	9.05	–	4.08	<0.001	0.33	8.89^b^
	Inexpressivity (Year 20)	−0.23	[−0.48, 0.02]	0.13	−0.22	−1.81	0.074	(0.30)	(1, 69)
	Avolition (Year 20)	−0.14	[−0.45, 0.17]	0.16	−0.13	−0.91	0.366		*p* = .004
	Emotional intelligence (Year 20)	0.26	[0.09, 0.43]	0.09	0.36	2.98	0.004		
Assessor-rated social and occupational functioning (SOFAS)									
1	Constant	65.50	[62.05, 68.95]	1.75	–	37.53	<0.001	0.53	
	Inexpressivity (Year 20)	0.04	[−0.21, 0.30]	0.13	0.02	0.34	0.731	(0.52)	
	Avolition (Year 20)	−1.45	[−1.73, −1.17]	0.14	−0.74	−10.31	<0.001		
2	Constant	38.29	[21.47, 55.12]	8.51	–	4.50	<0.001	0.56	10.64^b^
	Inexpressivity (Year 20)	0.09	[−0.16, 0.34]	0.13	−0.05	0.69	0.493	(0.55)	(1, 136)
	Avolition (Year 20)	−1.29	[−1.57, −1.00]	0.14	−0.66	−8.92	<0.001		*p* = .001
	Emotional intelligence (Year 20)	0.27	[0.11, 0.44]	0.08	0.21	3.26	0.001		
Self-rated functioning in independent living (WHODAS)^a^									
1	Constant	18.51	[15.93, 21.09]	1.30	–	14.22	<0.001	0.18	
	Inexpressivity (Year 20)	−0.28	[−0.50, −0.06]	0.11	−0.28	−2.53	0.013	(0.16)	
	Avolition (Year 20)	0.52	[0.30, 0.74]	0.11	0.52	4.71	<0.001		
2	Constant	23.90	[9.36, 38.43]	7.33	–	3.26	0.002	0.18	0.56
	Inexpressivity (Year 20)	−0.29	[−0.51, −0.07]	0.11	−0.29	−2.60	0.011	(0.16)	(1, 102)
	Avolition (Year 20)	0.49	[0.26, 0.72]	0.12	0.49	4.15	<0.001		*p* = .457
	Emotional intelligence (Year 20)	−0.05	[−0.20, 0.09]	0.07	−0.08	−0.75	0.457		

SSPA = Social Skills Performance Assessment; SOFAS = Social and Occupational Functioning Assessment Scale; WHODAS = World Health Organization Disability Assessment Schedule.

a Higher scores indicate greater functional impairment.

b Indicates statistically significant findings that persist after applying the FDR correction.

## Appendix A. Supplementary data

Supplementary data to this article can be found online at https://doi.org/10.1016/j.schres.2025.01.005.
