# Supplementary material for: Emotional intelligence as a predictor of functional outcomes in psychotic disorders
Source: Schizophr Res. Author manuscript; Available in PMC 2026 May 16. (PMC13179705; doi:10.1016/j.schres.2025.01.005)
Supplement: 1 [file NIHMS2170156-supplement-1.docx]

**Supplementary Materials**

| ***Supplementary Table 1.*** *Attrition analyses: Group differences in baseline demographics, diagnostic status, and clinical variables for participants included and not included in the current sample* | | | | | | | | |
| --- | --- | --- | --- | --- | --- | --- | --- | --- |
|  | Psychosis Group | | | Never-Psychotic Group | | | | |
| Characteristic | Included in sample  (N = 143) | Absent from sample  (N = 485) | Group Differences | | Included in sample  (N = 181) | Absent from sample  (N = 80) | | Group Differences |
|  | ***M* (*SD*) / n (%)** | ***M* (*SD*) / n (%)** |  | | ***M* (*SD*) / n (%)** | ***M* (*SD*) / n (%)** | |  |
| Age | 28.00 (7.94) | 30.90 (10.03) | *d* = .30** | | 51.44 (9.09) | 49.92 (8.61) | | *d* = .17 |
| Gender (Male) | 60 (42%) | 203 (42%) | z = 1.22 | | 85 (47%) | 30 (38%) | | *z =* -.21 |
| Race (White) | 119 (83%) | 381 (79%) | z = 0.02 | |  |  | | *z* = 1.42 |
| Diagnosis |  |  | *x^2^* = .96 | |  |  | |  |
| SSD | 41 (29%) | 142 (29%) |  | |  |  | |  |
| BP with psychotic features | 34 (24%) | 99 (20%) |  | |  |  | |  |
| MDD with psychotic features | 20 (14%) | 86 (18%) |  | |  |  | |  |
| Drug-induced psychosis | 7 (5%) | 21 (4%) |  | |  |  | |  |
| Other | 41 (29%) | 137 (28%) |  | |  |  | |  |
| Inexpressivity | 8.25 (9.07) | 6.54 (7.34) | *d* = .22* | | 0.86 (2.63) | 1.08 (3.09) | | *d =* 0.17 |
| Avolition | 10.28 (7.82) | 9.13 (9.13) | *d* = 0.11 | | 2.79 (3.89) | 2.86 (3.44) | | *d =* -0.08 |
| Positive/Reality Distortion | 10.64 (9.36) | 11.68 (9.49) | *d* = -0.09 | | 0.08 (.55) | 0.08 (.35) | | *d =* -0.02 |
| Disorganization | 7.06 (6.69) | 6.58 (6.27) | *d* = 0.10 | | 0.78 (2.30) | 1.23 (2.51) | | *d =* 0.02 |
| SSD = Schizophrenia spectrum disorder; BP = Bipolar disorder; MDD = Major depressive disorder  *d* = Cohen’s *d*;  *z* = Wald z-test; *x^2^* = chi-square test  * *p* < .05 *** p <* .001 | | | | | | |  | |

| ***Supplementary Table 2.*** *Correlations between emotional intelligence and functional outcomes in the psychosis group for those without missing data (Used for Steiger’s Z-tests)* | | | | |
| --- | --- | --- | --- | --- |
| N = 59 | Emotional Intelligence  (Year 20) | SSPA  (Year 25) | SOFAS  (Year 25) | WHODAS (Year 25) |
| Emotional Intelligence (Year 20) |  | <.001 | <.001 | .488 |
| SSPA (Year 25) | .47 |  | <.001 | .307 |
| SOFAS (Year 25) | .59 | .42 |  | .001 |
| WHODAS (Year 25) | -.09 | -.14 | -.41 |  |
| Highlighted cells indicate correlation coefficients. Non-highlighted cells indicate associated p-values. | | | | |

**Group status as a moderator of the relation between emotional intelligence and functioning, controlling for demographic variables**

We found group status (psychosis vs. never-psychotic) to significantly moderate the association between emotional intelligence and social performance five years later after controlling for education attainment and employment status (at both Year 20 and Year 25), *b* = .25, 95% CI [.08, .42], *t*(166) = 2.92, *p* = .004, β = .29. Specifically, the positive association between emotional intelligence and social performance was stronger for the psychosis group (*b* = .34, 95% CI [.22, .46], *t*(166) = 5.74, *p* < .001, β = .53) than for the never-psychotic group (*b* = .09, 95% CI [-.04, .22], *t*(166) = 1.41, *p* = .161, β = .14).

We found group status to significantly moderate the association between emotional intelligence and social and occupational functioning five years later after controlling for age and education attainment, *b* = .31, 95% CI [.05, .56], *t*(317) = 2.38, *p* = .018, β = .15 Specifically, the positive association between emotional intelligence and social performance was stronger for the psychosis group (*b* = .61, 95% CI [.44, .77], *t*(317) = 7.35, *p* < .001, β = .38) than for the never-psychotic group (*b* = .30, 95% CI [.10, .50], *t*(317) = 3.00, *p* = .003, β = .19).

We did not find group status to be a significant moderator of the association between emotional intelligence and self-reported functioning in independent living five years later after controlling for education attainment and employment status (at both Year 20 and Year 25), *b* = -.06, 95% CI [-.23, .10], *t*(259) = -.78, *p* = .437, β = -.07. Emotional intelligence was not found to significantly predict functioning in independent living for either those in the psychosis group (*b* = -.08, 95% CI [-.20, .03], *t*(259) = -1.48, *p* = .140, β = -.12) or those in the never-psychotic group (*b* = -.02, 95% CI [-.14, .10], *t*(259) = -.33, *p* = .745, β = -.03).

**Emotional intelligence as a predictor of functional outcomes controlling for negative symptoms and demographic variables**

Supplementary Tables 3, 4, and 5 display the hierarchical regression models for the psychosis group, taking into account age, education attainment, and employment status at Year 20 and Year 25. Age was only significantly associated with social and occupational functioning and thus only controlled for in that model. We did not control for employment status for the social and occupational functioning model, since this variable is accounted for in the outcome.

| ***Supplementary Table 3.*** *Regression coefficients for predictors of social performance controlling for education attainment and employment status* | | | | | | | | | |
| --- | --- | --- | --- | --- | --- | --- | --- | --- | --- |
| **Block** | **Variable** | **b** | **95% CI** | **SE** | $\boldsymbol{\beta}$ | ***t*** | ***p*** | **R^2^**  **(Adj. R^2^)** | $\Delta$**F-statistic**  **(df1, df2)** |
| **1** | Constant | 60.64 | [52.85, 68.44] | 3.91 | – | 15.53 | <.001 | .26  (.20) |  |
|  | Inexpressivity (Year 20) | -.26 | [-.54, .01] | .14 | -.25 | -1.91 | .060 |  |  |
|  | Avolition (Year 20) | -.36 | [-.72, .00] | .18 | -.32 | -1.98 | .052 |  |  |
|  | Education (Year 20) | .67 | [-.69, 2.04] | .68 | .11 | .98 | .330 |  |  |
|  | Employment (Year 20) | .27 | [-3.01, 3.54] | 1.64 | .02 | .16 | .872 |  |  |
|  | Employment (Year 25) | .33 | [-2.86, 3.52] | 1.60 | .03 | .21 | .838 |  |  |
| **2** | Constant | 31.22 | [10.51, 51.94] | 10.37 | – | 3.01 | .004 | .35  (.29) | 9.20  (1, 66)  *p =* .003 |
|  | Inexpressivity (Year 20) | -.25 | [-.51, .01] | .13 | -.23 | -1.89 | .063 |  |  |
|  | Avolition (Year 20) | -.19 | [-.55, .17] | .18 | -.17 | -1.07 | .289 |  |  |
|  | Education (Year 20) | .51 | [-.79, 1.80] | .65 | .08 | .78 | .437 |  |  |
|  | Employment (Year 20) | 1.30 | [-1.87, 4.46] | 1.59 | .12 | .82 | .416 |  |  |
|  | Employment (Year 25) | .39 | [-2.62, 3.40] | 1.51 | .03 | .26 | .796 |  |  |
|  | Emotional Intelligence (Year 20) | .27 | [.09, .45] | .09 | .39 | 3.03 | .003 |  |  |
|  | | | | | | | | | |

| ***Supplementary Table 4.*** *Regression coefficients for predictors of assessor-rated social and occupational functioning controlling for age and education attainment* | | | | | | | | | |
| --- | --- | --- | --- | --- | --- | --- | --- | --- | --- |
| **Block** | **Variable** | **b** | **95% CI** | **SE** | $\boldsymbol{\beta}$ | ***t*** | ***p*** | **R^2^**  **(Adj. R^2^)** | $\Delta$**F-statistic**  **(df1, df2)** |
| **1** | Constant | 50.54 | [38.61, 62.46] | 6.03 | – | 8.38 | <.001 | .57  (.56) |  |
|  | Inexpressivity (Year 20) | .00 | [-.25, .24] | .13 | .00 | -.04 | .970 |  |  |
|  | Avolition (Year 20) | -1.30 | [-1.58, -1.02] | .14 | -.67 | -9.20 | <.001 |  |  |
|  | Age (Year 20) | .19 | [-.05, .42] | .12 | .09 | 1.58 | .117 |  |  |
|  | Education (Year 20) | 2.29 | [.99, 3.58] | .66 | .21 | 3.48 | <.001 |  |  |
| **2** | Constant | 29.31 | [10.29, 48.33] | 9.61 | – | 3.05 | .003 | .60  (.58) | 7.79  (1, 134)  *p* = .006 |
|  | Inexpressivity (Year 20) | .04 | [-.21, .28] | .12 | .02 | .30 | .768 |  |  |
|  | Avolition (Year 20) | -1.19 | [-1.47, -.90] | .14 | -.61 | -8.22 | <.001 |  |  |
|  | Age (Year 20) | .17 | [-.06, .40] | .12 | .08 | 1.47 | .145 |  |  |
|  | Education (Year 20) | 2.01 | [.73, 3.30] | .65 | .18 | 3.11 | .002 |  |  |
|  | Emotional Intelligence (Year 20) | .23 | [.07, .39] | .08 | .17 | 2.79 | .006 |  |  |
|  | | | | | | | | | |

| ***Supplementary Table 5.*** *Regression coefficients for predictors of self-reported functioning in independent living controlling for education attainment and employment status* | | | | | | | | | |
| --- | --- | --- | --- | --- | --- | --- | --- | --- | --- |
| **Block** | **Variable** | **b** | **95% CI** | **SE** | $\boldsymbol{\beta}$ | ***t*** | ***p*** | **R^2^**  **(Adj. R^2^)** | $\Delta$**F-statistic**  **(df1, df2)** |
| **1** | Constant | 10.16 | [4.04, 16.28] | 3.08 | – | 3.29 | .001 | 28  (.25) |  |
|  | Inexpressivity (Year 20) | -.29 | [-.50, -.08] | .10 | -.29 | -2.74 | .007 |  |  |
|  | Avolition (Year 20) | .40 | [.15, .64] | .12 | .40 | 3.19 | .002 |  |  |
|  | Education (Year 20) | .57 | [-.48, 1.62] | .53 | .10 | 1.08 | .282 |  |  |
|  | Employment (Year 20) | -.33 | [-2.79, 2.12] | 1.24 | -.03 | -.27 | .787 |  |  |
|  | Employment (Year 25) | 4.05 | [1.72, 6.37] | 1.17 | .39 | 3.45 | <.001 |  |  |
| **2** | Constant | 11.92 | [-3.98, 27.82] | 8.01 | – | 1.49 | .140 | .28  (.24) | .06  (1, 99)  *p* = .812 |
|  | Inexpressivity (Year 20) | -.29 | [-.50, -.08] | .11 | -.29 | -2.73 | .008 |  |  |
|  | Avolition (Year 20) | .39 | [.14, .65] | .13 | .39 | 3.07 | .003 |  |  |
|  | Education (Year 20) | .57 | [-.48, 1.63] | .53 | .10 | 1.08 | .281 |  |  |
|  | Employment (Year 20) | -.37 | [-2.86, 2.11] | 1.25 | -.04 | -.30 | .768 |  |  |
|  | Employment (Year 25) | 4.02 | [1.68, 6.37] | 1.18 | .39 | 3.41 | <.001 |  |  |
|  | Emotional Intelligence (Year 20) | -.02 | [-.15, .12] | .07 | -.02 | -.24 | .812 |  |  |
|  | | | | | | | | | |

| ***Supplementary Table 6.*** *Regression coefficients for predictors of functional outcomes in the psychosis group: Emotional intelligence and negative symptoms at Year 20 and Year 25* | | | | | | | | |  |
| --- | --- | --- | --- | --- | --- | --- | --- | --- | --- |
| **Block** | **Variable** | **b** | **95% CI** | **SE** | $\boldsymbol{\beta}$ | ***t*** | ***p*** | **R^2^**  **(Adj. R^2^)** | $\Delta$**F-statistic**  **(df1, df2)** |
| ***Social Performance (SSPA)*** | | | | | | | |  |  |
| **1** | Constant | 63.83 | [60.12, 67.53] | 1.86 | – | 34.40 | <.001 | .27  (.22) |  |
|  | Inexpressivity (Year 20) | -.09 | [-.44, .25] | .17 | -.09 | -.54 | .592 |  |  |
|  | Avolition (Year 20) | -.26 | [-.70, .19] | .22 | -.22 | -1.15 | .254 |  |  |
|  | Inexpressivity (Year 25) | -.23 | [-.65, .18] | .21 | -.18 | -1.13 | .263 |  |  |
|  | Avolition (Year 25) | -.13 | [-.58, .32] | .22 | -.11 | -.59 | .557 |  |  |
| **2** | Constant | 38.89 | [19.66, 58.12] | 9.63 | – | 4.04 | <.001 | .34  (.29) | 6.95  (1, 64)  *p* = .011 |
|  | Inexpressivity (Year 20) | -.10 | [-.43, .22] | .16 | -.10 | -.63 | .531 |  |  |
|  | Avolition (Year 20) | -.12 | [-.56, .32] | .22 | -.11 | -.55 | .582 |  |  |
|  | Inexpressivity (Year 25) | -.22 | [-.61, .18] | .20 | -.17 | -1.08 | .283 |  |  |
|  | Avolition (Year 25) | -.02 | [-.46, .42] | .22 | -.02 | -.09 | .931 |  |  |
|  | Emotional Intelligence (Year 20) | .24 | [.06, .42] | .09 | .34 | 2.64 | .011 |  |  |
| ***Assessor-Rated Social and Occupational Functioning (SOFAS)*** | | | | | | | |  |  |
| **1** | Constant | 73.22 | [70.30, 76.14] | 1.47 | – | 49.65 | <.001 | .76  (.76) |  |
|  | Inexpressivity (Year 20) | -.08 | [-.36, .20] | .14 | -.04 | -.58 | .565 |  |  |
|  | Avolition (Year 20) | -.51 | [-.83, -.19] | .16 | -.25 | -3.17 | .002 |  |  |
|  | Inexpressivity (Year 25) | .31 | [-.01, .63] | .16 | .14 | 1.90 | .059 |  |  |
|  | Avolition (Year 25) | -1.56 | [-1.90, -1.21] | .17 | -.71 | -9.00 | <.001 |  |  |
| **2** | Constant | 58.34 | [44.33, 72.36] | 7.08 | – | 8.25 | <.001 | .77  (.76) | 4.62  (1, 114)  *p =* .034 |
|  | Inexpressivity (Year 20) | -.07 | [-.35, .20] | .14 | -.04 | -.54 | .593 |  |  |
|  | Avolition (Year 20) | -.45 | [-.77, -.14] | .16 | -.22 | -2.84 | .005 |  |  |
|  | Inexpressivity (Year 25) | .32 | [.00, .63] | .16 | .14 | 1.99 | .049 |  |  |
|  | Avolition (Year 25) | -1.50 | [-1.84, -1.16] | .17 | -.68 | -8.68 | <.001 |  |  |
|  | Emotional Intelligence (Year 20) | .15 | [.01, .28] | .07 | .11 | 2.15 | .034 |  |  |
| ***Self-Rated Functioning in Independent Living (WHODAS)^1^*** | | | | | | | |  |  |
| **1** | Constant | 17.16 | [14.28, 20.03] | 1.45 | – | 11.85 | <.001 | .20  (.17) |  |
|  | Inexpressivity (Year 20) | -.19 | [-.50, .13] | .16 | -.19 | -1.17 | .243 |  |  |
|  | Avolition (Year 20) | .31 | [-.02, .64] | .17 | .32 | 1.89 | .062 |  |  |
|  | Inexpressivity (Year 25) | -.11 | [-.47, .24] | .18 | -.10 | -.63 | .531 |  |  |
|  | Avolition (Year 25) | .27 | [-.08, .63] | .18 | .25 | 1.55 | .125 |  |  |
| **2** | Constant | 26.01 | [10.61, 41.41] | 7.75 | – | 3.36 | .001 | .21  (.17) | 1.35  (1, 86)  *p* = .248 |
|  | Inexpressivity (Year 20) | -.20 | [-.52, .12] | .16 | -.20 | -1.26 | .213 |  |  |
|  | Avolition (Year 20) | .28 | [-.05, .61] | .17 | .29 | 1.68 | .097 |  |  |
|  | Inexpressivity (Year 25) | -.12 | [-.47, .24] | .18 | -.10 | -.65 | .520 |  |  |
|  | Avolition (Year 25) | .24 | [-.11, .60] | .18 | .22 | 1.36 | .178 |  |  |
|  | Emotional Intelligence (Year 20) | -.09 | [-.24, .06] | .07 | -.13 | -1.16 | .248 |  |  |
| SSPA = Social Skills Performance Assessment; SOFAS = Social and Occupational Functioning Assessment Scale; WHODAS = World Health Organization Disability Assessment Schedule  ^1^ = Higher scores indicate greater functional impairment | | | | | | | | | |
